# Severe SARS-CoV-2 and subsequent fungal infections after CAR T-cell therapy for relapsed/refractory multiple myeloma: a challenging and happy ending fight

**DOI:** 10.1016/j.lrr.2023.100399

**Published:** 2023-11-27

**Authors:** Claudia Ielo, Francesca Fazio, Serena Rocchi, Ilaria Rizzello, Katia Mancuso, Elena Zamagni, Michele Cavo, Maria Teresa Petrucci

**Affiliations:** a Hematology Unit, Department of Translational and Precision Medicine, Azienda Policlinico Umberto I, Sapienza University of Rome, Italy; b Seràgnoli Institute of Hematology, Department of Experimental, Diagnostic, and Specialty Medicine, Bologna University School of Medicine, S. Orsola Malpighi Hospital, Bologna, Italy

**Keywords:** Multiple myeloma, Cellular therapies, Infectious complications

## Abstract

Chimeric antigen receptor (CAR) T-cells have unveiled a promising therapeutic horizon for relapsed/refractory multiple myeloma (R/R MM). Nevertheless, immune impairment induced by cellular therapies, previous treatments and MM itself could promote infectious events. COVID-19 could evolve into a life-threating infection in R/R MM patients who often have suboptimal responses to SARS-CoV-2 vaccines. Here, we describe a case of severe and long-lasting COVID-19 pneumonia after CAR T-cell therapy for R/R MM requiring a complex clinical management. Long-term infectious complications in MM patients undergoing CAR T-cells should be taken into consideration as they could counteract the efficacy of this new treatment.

## 1 Background

Multiple myeloma (MM) patients whose disease is refractory to CD38-targeting monoclonal antibodies, proteasome inhibitors and immunomodulatory drugs (named triple refractory) have limited treatment options and display a poor prognosis [1]. Ide-cel (idecabtagene vicleucel) and cilta-cel (ciltacabtagene autoleucel), the first chimeric antigen receptor (CAR) T-cells targeting the B cell maturation antigen (BCMA), proved highly effective in the setting of triple-refractory MM patients [2]. Nevertheless, CAR-T cells recipients are exposed to a high risk of infections based on data from CD-19 CAR-T studies and increasing data on infective complications in R/R MM patients undergoing cellular immunotherapies are available [3]. In the context of the COVID-19 pandemic, findings from a retrospective observational multicentric study suggests this vulnerability could translate into a life-threatening course of the SARS-CoV-2 infection [4]. Appropriate management of COVID-19 in lymphodepleted patients is challenging and has not been defined yet. Here, we report the case of a R/R MM patient who was successfully treated for a severe COVID-19 pneumonia developing after CAR-T cell therapy.

## 2 Case presentation

A 51-year-old woman with anemia and rib fractures was diagnosed with IgG-k MM in 2012. Serum monoclonal component was 6.3 g/dl and Bence Jones proteinuria was 2.3 g/24 h. Cytogenetic analysis showed amplification of 1q21 and, according to the International Staging System (ISS) and Revised-ISS, the disease was classified as stage I and I, respectively.

After enrollment in the EMN02 trial, the patient received 4 induction cycles with bortezomib, cyclophosphamide and dexamethasone, followed by tandem autologous stem cell transplantation and 2 consolidation cycles with bortezomib, lenalidomide and dexamethasone. Complete remission was achieved at the end of consolidation, and she started maintenance therapy with lenalidomide, according to clinical trial. Unexpectedly, in 2020 she experienced clinical relapse. Subsequent treatment consisted of 8 cycles of induction therapy with carfilzomib, pomalidomide and dexamethasone followed by maintenance with pomalidomide within the EMN11 trial, leading to partial remission. After a few months, the patient experienced biochemical relapse and third line therapy with daratumumab, lenalidomide and dexamethasone was started. Due to refractoriness to third-line therapy, the patient was enrolled in the CARTITUDE-4 trial [5] and randomized to receive CAR T-cell therapy in December 2021. On third day post-infusion, grade 2 cytokine release syndrome developed and resolved with tocilizumab and steroids. Minimal residual disease (MRD) negativity has been achieved 6 months after infusion.

**Fig. 1:**
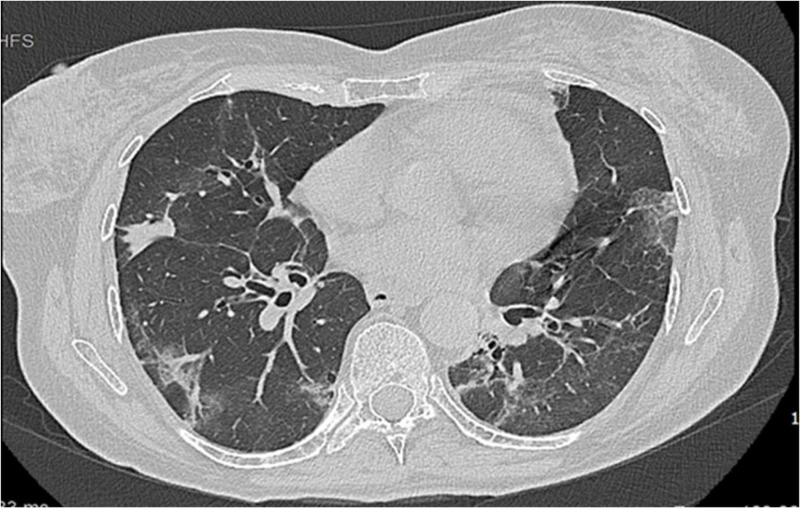
Chest CT scan depicting bilateral SARS-CoV-2 interstitial pneumonia.

In July 2022 the patient contracted SARS-CoV-2 infection presenting with dry cough and low-grade fever. She had been previously vaccinated against SARS-CoV-2 with two doses of the mRNA vaccine BNT162b2, with no serological response, before undergoing CAR T-cell therapy and received a third boost in March 2022. Despite antiviral therapy with molnupiravir, cough worsened, and bilateral interstitial pneumonia was detected by CT scan (Fig. 1) on admission to hospital. Blood tests showed lymphopenia (800 cells/µl), hypogammaglobulinemia (200 mg/dl) and high levels of inflammatory markers (CRP 38,000 µgr/l and IL-6 38 pg/ml). Remdesivir and tixagevimab-cilgavimab did not improve her clinical condition which was exacerbated by pulmonary embolism and required high-flow oxygen therapy. Viral genotyping revealed non BA.2 omicron variant, viremia resulted positive and antinucleocapsid antibodies were not identified. Analysis of lymphocyte subpopulation displayed B-cell aplasia (10 cells/µl), CD4+ T cells (200 cells/µl) and NK cells (37 cells/µl) deficiency. Eventually, hyper immune plasma was infused obtaining gradual resolution of opacities and improvement of respiratory failure. Soon after pulmonary aspergillosis superimposed and resolved with a two-month course of isavuconazole. Intravenous immunoglobulin G therapy (IVIG) at a dosage of 400 mg/kg has been administered weekly during antifungal treatment in order to reach normal IgG levels (≥ 700 mg/dl). In December 2022 SARS-CoV-2 clearance was achieved and the patient was discharged after a five-month hospitalization.

Thus far, the patient is still in complete remission, with bone marrow MRD negativity, more than one year after CAR-T infusion. After the resolution of infectious events, the patient received IVIG every three weeks until April 2023. She is currently receiving only antiviral prophylaxis for Varicella-Zoster and Herpes Simplex Viruses and antibacterial prophylaxis for Pneumocystis jirovecii pneumonia. She had good physical recovery and was not affected by any post COVID-19 sequelae limiting her daily activities (Karnofsky Performance Status ≥ 90 %). Complete blood count is normal except for mild normocytic anemia (10–11 gr/dl) and IgG levels are >700 mg/dl.

## 3 Discussion

Patients receiving CAR-T cells targeting B-lineage surface antigens have significant B -cell aplasia, requiring IVIG supplementation and may also develop delayed and prolonged cytopenias, which might limit humoral response to viral infections. Infectious complications following CAR T-cell therapy have been mostly described in patients affected by B lymphoid malignancies treated with CD19-targeting cellular products. Their incidence in prospective clinical trials and retrospective analyses is up to 56 % and 60 %, respectively. Bacterial complications are the most common, accounting for up to 50 % of the infections, and their typically onset is within 30 days after CAR T-cell infusion, during the period of high-grade neutropenia [3].

By contrast, R/R MM patients receiving BCMA-targeting CAR T-cell seem to have a different pattern of infections, according to recent retrospective studies. Joshula et al. [6] analyzed a cohort of 32 R/R MM patients and, within 180 days after BCMA CAR T-cell infusion, 17 patients (53 %) developed a total of 23 infections; 13 out of 23 (57 %) were mild/moderate viral infections. Kambhampati et al. [7] showed 47 infections in 29 (53 %) of 55 patients with R/R MM up to 1 year after BCMA CAR T-cell infusion. Viral infections were 25 (53 %) showing a mild/moderate severity and were caused by respiratory viruses. In a heterogeneous cohort of 68 R/R MM patients who were treated with anti-CD19 combined with anti-BCMA CAR T-cell therapy or with anti-BCMA CAR T-cell therapy alone, Zhou et al. [8] observed a total of 43 infections in 26 patients (38 %) within 12 months after CAR T-cell infusion. Most infectious events were bacterial infections (81.4 %), followed by fungal infections (11.6 %) and viral infections (7 %). The authors hypothesized an underestimation of viral infections due to their auto limiting course and their mild/moderate severity, and identified several prior lines of treatment, longer duration of neutropenia after infusion, severe CRS and poor treatment response as independent risk factors associated with infections [8].

The outcomes of patients who developed COVID-19 infection after CAR T-cell therapy have been explored in few studies. Data from the European registry on CAR T-cell recipients with symptomatic SARS-CoV-2 infection, demonstrated a prevalence of COVID-19 of 4.8 %, based on the total number of CAR T-cell recipients recorded by participating centers in 2020 (*n* = 17 of 353) [4]. Reported overall mortality was 50 %, highlighting the need for prevention of infection in these patients, and was higher compared to that observed in patients who had undergone hematopoietic stem cell transplantation. In addition, about half of the patients developed COVID-19 within 6 months post CAR T-cell therapy [4]. Moreover, emerging researches are corroborating the evidence that CAR-T cell recipients have limited humoral response to SARS-CoV-2 vaccinations. Aleissa et al. [9] evaluated seropositivity rate in 50 patients who received CD19 or BCMA CAR T-cell therapy. Most patients (*n* = 32, 64 %) received at least one vaccination post CAR T-cell therapy and 64 % of the patients had positive anti-spike titers. Regrettably, 35 % of the patients who were vaccinated with three or more doses were unable to mount a humoral response [9]. Our patient received two doses of mRNA vaccine before CAR-T without serological response and a third boost after CAR-T but still contracted COVID-19 suggesting responses to the vaccinations were inadequate. Notably, MM patients have an intrinsic compromised humoral immunity and delayed reconstitution of normal plasma cells level was observed after anti-BCMA CAR T-cell therapy [10]. Long-term hypogammaglobulinemia is a common on-target off-tumor toxicity in CAR T-cell recipients because CAR-T cells target B-lineage surface antigens such as CD19, CD22 and BCMA [11]. Immunoglobulin levels should be measured routinely and IVIG or subcutaneous immunoglobulin replacement can be considered to maintain serum levels >400 mg/dl to reduce the risk of sinopulmonary infections [12]. Besides hypogammaglobulinemia related to B-cell aplasia, our patient displayed deficiency of cellular immunity which could explain the severe course of SARS-CoV2.

To date there is limited evidence on optimal therapeutic strategies against COVID-19 in immunocompromised patients. Combined treatments against SARS-CoV-2 including passive immunotherapy (convalescent plasma and/or monoclonal antibodies) and antivirals can be effective therapies in B-cell depleted patients [13,14]. Pre-exposure prophylaxis with tixagevimab-cilgavimab, a monoclonal-antibody combination, proved effective in participants who were at increased risk for an inadequate response to COVID-19 vaccinations with a relative risk reduction of 82.8 % [15]. Hence, tixagevimab-cilgavimab was authorized in 2021 by FDA (Food and Drug Administration) and subsequently by EMA (European Medicines Agency) for prevention of COVID-19 in immunocompromised patients. In January 2023 its authorization was revoked by FDA as new omincron subvariants were prevalent in the United States at that time [12].

As shown by our case, CAR T-cell recipients can be affected by a complex immune impairment exposing them to severe SARS-CoV-2 infections. Careful monitoring of immune reconstitution after CAR-T cell therapy is crucial in this hematological population and further investigations are warranted in order to identify tailored therapeutic strategies against COVID-19.

## Author contributions

Claudia Ielo, Francesca Fazio, Elena Zamagni, Michele Cavo and Maria Teresa Petrucci conceptualized the manuscript. Claudia Ielo and Francesca Fazio wrote the first draft of the manuscript. Elena Zamagni, Michele Cavo and Maria Teresa Petrucci supervised the research. All authors were involved in the revision and editing of the manuscript.

## Funding information

The authors did not receive funding from any organization for the submitted manuscript.

## Informed consent

Written informed consent was obtained from the patient, including permission for the publication of the image.

## Declaration of Competing Interest

No potential conflict of interest was reported by the authors.

## Data Availability

The data are available from the corresponding author upon reasonable request.
